# The mechanism of water pollutant photodegradation by mixed and core–shell WO_3_/TiO_2_ nanocomposites†

**DOI:** 10.1039/d3ra01582c

**Published:** 2023-04-25

**Authors:** Abdisa Habtamu, Masaki Ujihara

**Affiliations:** a Graduate Institute of Applied Science and Technology, National Taiwan University of Science and Technology 43 Keelung Road 10607 Taipei Taiwan masaki.ujihara@mail.ntust.edu.tw

## Abstract

Environmental pollution is one of the biggest concerns in the world today, and solar energy-driven photocatalysis is a promising method for decomposing pollutants in aqueous systems. In this study, the photocatalytic efficiency and catalytic mechanism of WO_3_-loaded TiO_2_ nanocomposites of various structures were analyzed. The nanocomposites were synthesized *via* sol–gel reactions using mixtures of precursors at various ratios (5%, 8%, and 10 wt% WO_3_ in the nanocomposites) and *via* core–shell approaches (TiO_2_@WO_3_ and WO_3_@TiO_2_ in a 9 : 1 ratio of TiO_2_ : WO_3_). After calcination at 450 °C, the nanocomposites were characterized and used as photocatalysts. The kinetics of photocatalysis with these nanocomposites for the degradation of methylene blue (MB^+^) and methyl orange (MO^−^) under UV light (365 nm) were analyzed as pseudo-first-order reactions. The decomposition rate of MB^+^ was much higher than that of MO^−^, and the adsorption behavior of the dyes in the dark suggested that the negatively charged surface of WO_3_ played an important role in adsorbing the cationic dye. Scavengers were used to quench the active species (superoxide, hole, and hydroxyl radicals), and the results indicated that hydroxyl radicals were the most active species; however, the active species were generated more evenly on the mixed surfaces of WO_3_ and TiO_2_ than on the core–shell structures. This finding shows that the photoreaction mechanisms could be controlled through adjustments to the nanocomposite structure. These results can guide the design and preparation of photocatalysts with improved and controlled activities for environmental remediation.

## Abstract 2

Mixed and core–shell WO_3_/TiO_2_ exhibited different photocatalytic activities due to their surface charges and charge separation behaviors.

## Graphical abstract

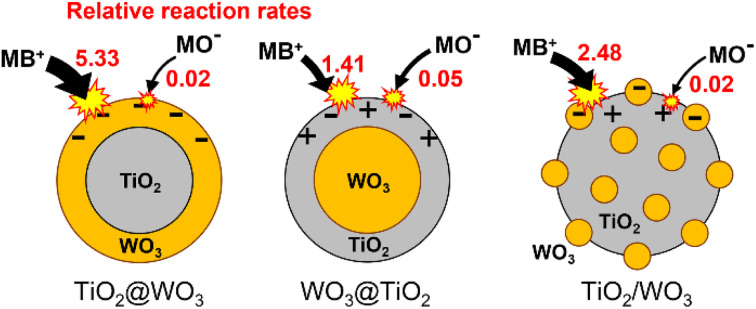

## 1. Introduction

Increasing wastewater discharges from various sources pose enormous environmental challenges worldwide.^1^ Due to rapid industrial growth, the environment has become highly contaminated with various organic and inorganic pollutants.^2,3^ Dyes are common hazardous organic contaminants in wastewater.^4^ They also impart color to the water and can produce harmful byproducts through chemical reactions.^5^ The long-term consumption of water containing dyes could harm the liver, central nervous, and digestive systems of humans.^6^ For this reason, many researchers are engaged in developing techniques to eliminate organic dyes from water systems.

Recently, various techniques, including using a green biochar/iron oxide composite,^7^ coated membranes,^8^ surfactant-modified biomass,^9^ coagulation,^10^ modified magnetic nanosorbents,^11,12^ hydrochar adsorption,^13,14^ and photocatalytic degradation,^15,16^ have been applied to remove both cationic and anionic dyes from water. Among these methods, semiconductor-based photocatalysis is thought to be the most promising method because it can convert a broad range of organic contaminants into less toxic compounds, including CO_2_, and H_2_O, without the use of expensive oxidants. With the aim of developing effective photocatalysts, various semiconductors have been examined individually or in combination with other materials. The modification of photocatalyst surfaces with other materials can improve the efficiency of photocatalysis.^17^ Combining semiconductors with metals can enhance charge separation.^18–21^ Elemental doping and combining different semiconductors^22,23^ can change the band gap of the resulting materials and induce charge separation. Among the photocatalysts, TiO_2_ has been widely investigated as a typical semiconductor photocatalyst^24,25^ due to its high photocatalytic activity, low price, physicochemical stability, nontoxicity, and environmental friendliness.^26^ Despite these advantages, the wide bandgap of TiO_2_ (3.20–3.35 eV) limits the use of light to the UV range, shows rapid charge recombination and has limited efficiency.^27^ To address these limitations, doping and combining TiO_2_ photocatalysts with narrow bandgap semiconductors are viable options. Semiconductors such as MoO_3_,^28^ Ag_2_CO_3_,^29^ ZnO,^30^ and WO_3_ (ref. 27) have been coupled with TiO_2_ to improve its photocatalytic efficiency under UV light. Among them, WO_3_ has attracted considerable amounts of attention due to its ability to absorb visible light (typically wavelengths <500 nm (ref. 31 and 32) and extended to >500 nm by the effects of oxygen vacancies^32^) WO_3_ is also stable in oxidative and acidic environments and has low cost and low toxicity.^33^ The crystal ionic radius of W^6+^ is close to that of T^4+^; therefore, W^6+^ can be easily introduced into the lattice of titania to replace Ti^4+^ and form W–O–Ti links, or it can be positioned at interstitial locations,^34,35^ which effectively induces lattice defects and increases the surface area of WO_3_-coupled TiO_2_. Moreover, WO_3_-coupled TiO_2_ shows enhanced O_2_ chemisorption on its surfaces,^36^ and this adsorbed oxygen improves charge separation. Thus, WO_3_-coupled TiO_2_ has emerged as a promising adsorbent and catalyst. However, based on the preparation methods and the nature of pollutants to be decomposed, different behaviors have been reported.^34,36–40^ Some studies have stated that WO_3_ doping boosted TiO_2_ photocatalytic activity, whereas others reported that it had the reverse effect. Various factors, such as the nature of the dopants and their concentrations, the nature of pollutants, the intensity of light and irradiation time, dissolved oxygen concentration, reaction temperature, pH, surface area, the quantity of catalyst, and the surface morphology of the catalysts,^41^ are now considered to have an impact on photocatalytic decomposition. For effective photocatalysts, WO_3_/TiO_2_ core–shell nanorods were developed.^42,43^ Mixed WO_3_/TiO_2_ composites were utilized.^27,36,40,44–48^ However, the comparative study among the core–shell and co-mixed structures, and the effects of structures on reaction mechanisms were not strongly reported. Therefore, we focused on the structural dependence for the dye decomposition.

During the photodecomposition process, adsorption of target compounds is a key first step to be considered.^49–51^ In this study, cationic and anionic dyes (MB^+^ and MO^−^) were used to analyze the adsorption process of target compounds on the surface of WO_3_-loaded TiO_2_. Then, 3 different types of WO_3_–TiO_2_ nanocomposites (a mixture of TiO_2_ and WO_3_ formed by the sol–gel reaction and core–shell structures of TiO_2_@WO_3_ and WO_3_@TiO_2_ prepared by a hydrothermal method) were examined as photocatalysts, in addition to the single-component photocatalysts (TiO_2_ and WO_3_). The photodegradation of model target compounds was analyzed in terms of both the adsorption kinetics and reaction mechanism. The results can help guide the further design of photocatalysts consisting of semiconductor nanocomposites.

## 2. Experimental section

### 2.1 Materials

Titanium(iv) isopropoxide (TTIP, ACROS ORGANICS, CHINA), isopropanol (≥99.8% (GC), Honeywell|Riedel-de Haën™, France), sodium tungstate oxide dihydrate (Na_2_WO_4_·2H_2_O, 99.0–101.0%, Thermo Fisher Scientific.), ethanol (≥99.9%, Honeywell|Fluka™, Germany), nitric acid (65% w/w), hydrochloric acid (37%, reagent grade), ethylene glycol (99.5%, ACROS ORGANICS), methylene blue (ACROS ORGANICS, INDIA), and methyl orange (ACROS ORGANICS, INDIA) were used without further purification. *Para*-benzoquinone (*p*-BQ) and ethylenediaminetetraacetic acid disodium salt (Na_2_-EDTA) were purchased from Sigma-Aldrich and Fisher Chemical, respectively. Ultrapure water (resistivity of 18.2 mΩ cm, Yamato, Japan) was used throughout the experiments.

### 2.2 Sol–gel synthesis of TiO_2_ nanoparticles

The TiO_2_ nanoparticles were prepared *via* a sol–gel approach.^52^ First, 6.0 mL of TTIP was mixed with 11.6 mL of isopropanol. The mixture was vigorously stirred for 1 h, and 14.6 mL of water was added with vigorous stirring. After aging for 24 h, the white precipitate that formed was filtered and thoroughly washed with water. Then, the residue was dried at 80 °C for 12 h and calcined at 450 °C for 2 h. The obtained white mass was ground into a powder with a mortar and pestle.

### 2.3 Hydrothermal synthesis of TiO_2_ nanoparticles

For comparison, TiO_2_ nanoparticles were synthesized by a hydrothermal method.^53^ First, 5.9 mL of TTIP was dissolved in 9.0 mL of ethylene glycol and stirred for 2 h. Then, the solution was transferred into a Teflon-lined autoclave, and 30.3 mL of water was added. The white slurry formed was heated at 220 °C for 6 h, and the resulting white precipitate was washed three times with water and twice with ethanol using centrifugation. Then, the white paste was dried at 80 °C for 12 h and calcined at 450 °C for 2 h. The obtained white mass was ground to a powder with a mortar and pestle. This powder was labeled hyd-TiO_2_.

### 2.4 Synthesis of WO_3_ nanoparticles by the sol–gel method

The sol–gel procedure for WO_3_ synthesis was adapted from a previous report.^23^ A powder of Na_2_WO_4_·2H_2_O (1.0 g) was dissolved in 15 mL of water with stirring. To the solution, 7 mL of 1.0 M HCl was slowly added under vigorous stirring. Then, the obtained light yellowish solution was heated to 80 °C for 1 h. After the suspension was cooled to ambient temperature, the light yellowish precipitate was separated using centrifugation and washed three times with water to remove residual NaCl and HCl. Then, the yellow paste was dried at 80 °C for 12 h and ground into a powder with a mortar and pestle.

### 2.5 Hydrothermal synthesis of WO_3_ nanoparticles

WO_3_ nanoparticles were also prepared by a hydrothermal method. A powder of Na_2_WO_4_·2H_2_O (2.0 g) was dissolved in 30.0 mL of water. Then, 10 mL of 5 N HNO_3_ was added to the solution with vigorous stirring at ambient temperature. The mixture was moved to an autoclave and heated to 220 °C for 6 h. After the mixture was cooled to room temperature, the precipitates were collected by centrifugation, washed 3 times with water followed by ethanol, and dried at 80 °C for 12 h. A yellowish mass was obtained after calcination at 450 °C for 2 h and was ground to a powder with a mortar and pestle. This powder was labeled hyd-WO_3_.

### 2.6 Sol–gel synthesis of WO_3_/TiO_2_ nanocomposites

Coprecipitation of WO_3_ and TiO_2_ was carried out for the preparation of the WO_3_/TiO_2_ nanocomposites. First, 6.0 mL of TTIP was mixed with 11.6 mL of isopropanol, and the mixture was vigorously stirred for 1 h. To control the ratio of WO_3_ in the nanocomposites, a powder of Na_2_WO_4_·2H_2_O (0.243 g for 5 wt%, 0.401 g for 8 wt%, and 0.512 g for 10 wt%) was dissolved in 14.6 mL of water and added to the TTIP solution with vigorous stirring. After the mixture was aged for 24 h, the precipitate was filtered and washed 3 times with water followed by ethyl alcohol. Finally, nanocomposites were obtained after drying at 80 °C for 12 h and calcination at 450 °C for 4 h, followed by crushing in a mortar. The single-component metal oxides (TiO_2_ and WO_3_) were denoted sol-TiO_2_ and sol-WO_3_, respectively.

### 2.7 Synthesis of core–shell TiO_2_@WO_3_

The core–shell nanocomposite TiO_2_@WO_3_ was produced with a hydrothermal method.^54^ The hydrothermally synthesized TiO_2_ (hereafter, called hyd-TiO_2_, 632.5 mg) was dispersed in a solution of Na_2_WO_4_·2H_2_O (100 mg of Na_2_WO_4_·2H_2_O dissolved in 30 mL of water) with stirring for 60 min; the final mass ratio of TiO_2_ : WO_3_ = 9 : 1. The resulting white suspension was treated with dropwise additions of 5 N HNO_3_ with vigorous stirring. Then, the suspension was transferred to a 50 mL Teflon-lined autoclave and heated at 220 °C for 6 h. The precipitate was washed using water and ethanol by centrifugation and dried at 80 °C for 12 h. Then, the TiO_2_@WO_3_ composite was obtained after calcination in air at 450 °C for 2 h, followed by crushing in a mortar.

### 2.8 Synthesis of core–shell WO_3_@TiO_2_

The reverse core–shell structure of TiO_2_@WO_3_ was also synthesized. First, 2.4 mL of TTIP and 10 mL of ethylene glycol were mixed and stirred for 2 h at ambient temperature. Next, a suspension of WO_3_ was prepared by sonicating 70.3 mg of hyd-WO_3_ in 28.9 mL of water for 60 min. These solutions and suspensions were mixed in a 50 mL Teflon-lined autoclave to make the final mass ratio of TiO_2_ : WO_3_ 9 : 1. Then, the obtained yellowish to white gel was heated at 220 °C for 6 h. After cooling, the product was washed using water and ethanol by centrifugation and dried for 12 h at 80 °C. Finally, a yellowish to white mass was calcined at 450 °C for 2 h, followed by crushing in a mortar.

### 2.9 Characterization

The morphology of the nanocomposites was observed with a field-emission scanning electron microscope (FESEM, JSM-7900F, JEOL LTD, Japan) at an acceleration voltage of 15 kV. Before SEM inspection, all samples were sputtered with Pt using a JEC-3000FC Auto Fine Coater (JEOL LTD, Japan). Elemental analysis was carried out using an energy dispersive X-ray (EDX) spectrometer equipped with an FESEM. The crystal structures were characterized using XRD (X-ray diffractometer, 2nd Gen D2 PHASER, Bruker) with Cu Kα radiation at an acceleration voltage of 30 kV and a current of 10 mA within the 2*θ* range from 10° to 80°. The presence and oxidation state of each element in the nanocomposites were determined using X-ray photoelectron spectroscopy (XPS, ULVAC PHI 5000 Versa Probe) using Al Kα monochromator (1486.6 eV) X-rays. A UV-VIS spectrophotometer (V-670, JASCO, Japan) was used to measure the absorption spectra of the organic dye solutions. UV-VIS diffuse reflectance spectroscopy (DRS) was conducted at a 45° irradiation angle with a UV-VIS spectrometer (SEC2000, ALS, Japan) with a light source from Ocean Optics DH-2000-BAL.

### 2.10 Adsorption analyses

The adsorption properties of the nanocomposites were analyzed by a batch process. A powder of the nanocomposite (4 mg) was dispersed in dye solutions (65 mL) of varying concentrations (1.0, 2.0, 4.0, 6.0, and 8.0 mg L^−1^). Then, the suspension was stirred at ambient temperature under dark conditions for 30 min to achieve adsorption equilibrium. After adsorption, the suspension was separated into a supernatant and precipitate (the nanocomposite absorbed some of the dye) by centrifugation (for 60 s at 6000 rpm at neutral pH and room temperature), and the free dye concentration was determined from the UV-VIS absorption spectra of the supernatant: absorbance at the *λ*_max_ of the dye (662 nm for MB^+^ and 464 nm for MO^−^) was compared to that of the original dye solution (see Fig. S1 in the ESI†). The quantity of dye adsorbed on each nanocomposite (*q*_e_) in mg g^−1^ was estimated according to eqn (1).1
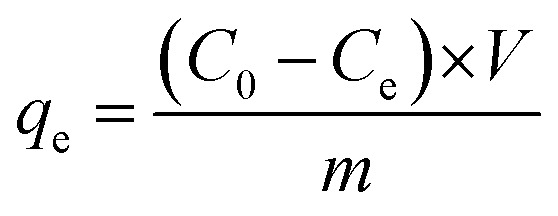
where *C*_0_ and *C*_e_ are the dye concentrations (ppm) before and after adsorption from the solution, *V* is the volume (L) of the dye solution (=0.065), and *m* is the mass (g) of the nanocomposite. The theoretical curves were fitted to data plots by the software (ORIGIN 2018) with *R*^2^ values.

### 2.11 Photocatalytic activity analyses

To evaluate photocatalytic activity, a powder of the nanocomposite (4 mg) was dispersed in 65 mL of a dye solution (2.0 ppm) under continuous stirring at room temperature. After 30 min in the dark, the dispersion was irradiated at 365 nm using an LED light source (LLS-365, Ocean Optics, Tokyo, Japan). Then, 2.0 mL of the dispersion was sampled at 10 min intervals and centrifuged for solid–liquid separation. The dye concentration of the supernatant was then measured at the *λ*_max_ of the dye. To confirm the reactive species, scavenger solutions (1 ppm of *p*-BQ, Na_2_-EDTA, and IPA) were used to scavenge superoxide radicals, holes, and hydroxyl radicals, respectively. The quantity (*Q*_d_) of dye degraded was estimated in mg g^−1^ by subtracting the free dye concentration at time *t* (*C*_*t*_, mg L^−1^) from the dye concentration before light irradiation. Then, the decomposed quantity of the dye was calculated using eqn (2).2
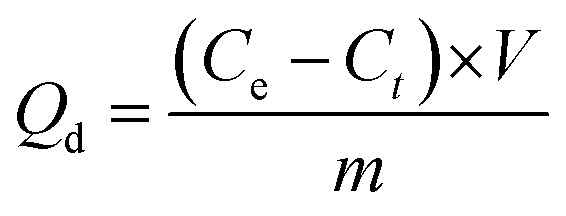
where *V* is the volume (L) of dye solution at the sampling time, and *m* is the mass (g) of catalysts.

The dye decomposition efficiency (%*D*) was also assessed using eqn (3).3
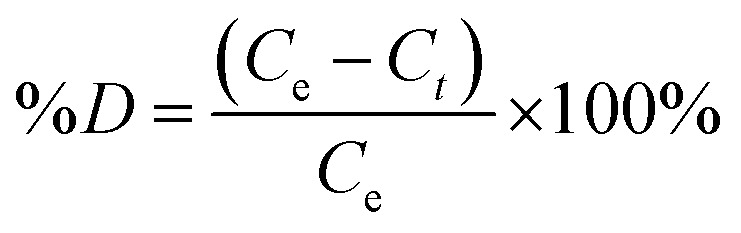


The decomposition kinetics were analyzed as pseudo first-order reactions using the Langmuir–Hinshelwood model^55^ and were plotted as ln(*C*_0_/*C*_*t*_) *vs.* the photoirradiation time (*t*, min), as in eqn (4).4
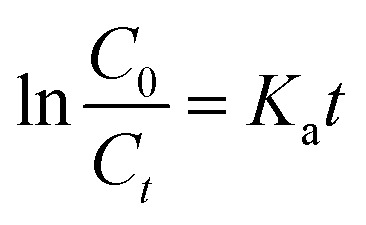
where *K*_a_ is the degradation rate constant (min^−1^). The theoretical curves were fitted to data plots by the software (ORIGIN 2018) with *R*^2^ values.

## 3. Results and discussion

### 3.1 Structure of nanocomposites

**Fig. 1:**
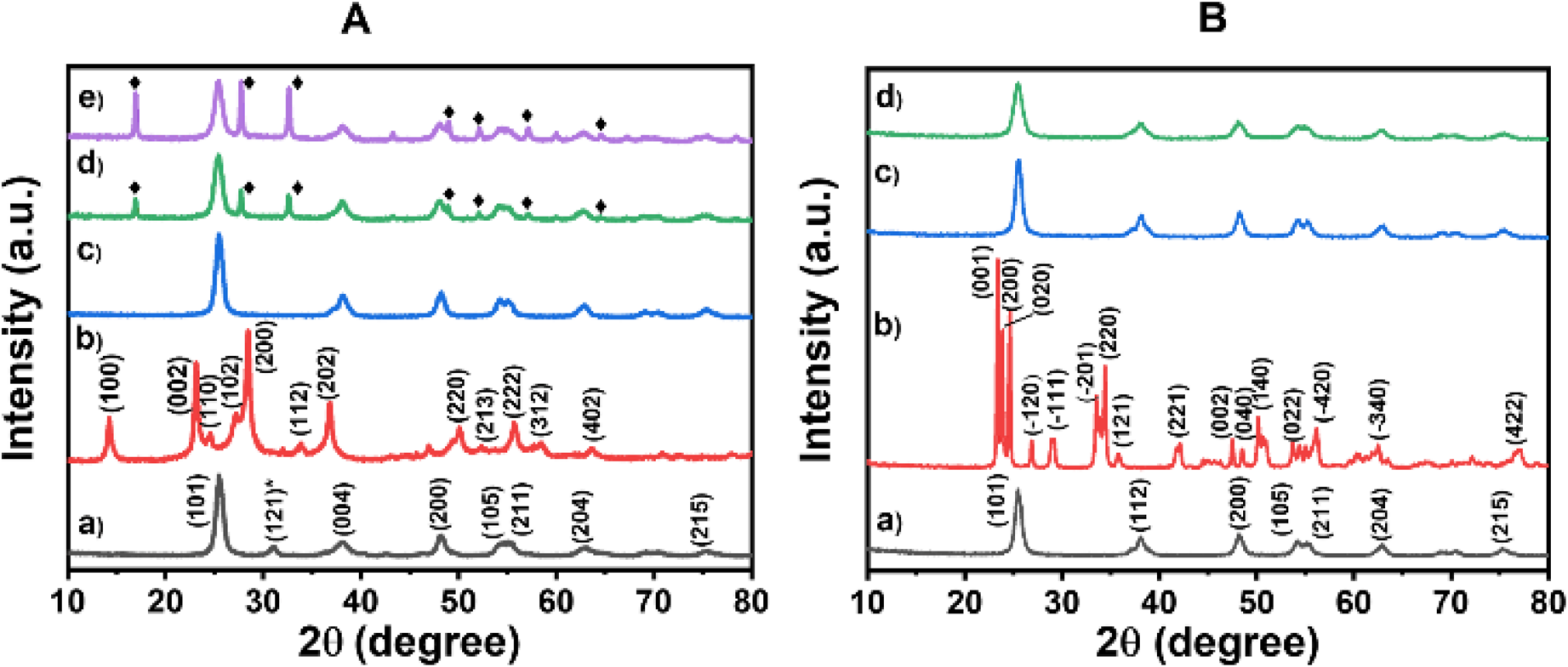
XRD results of (A) the sol–gel prepared (a) sol-TiO_2_, (b) sol-WO_3_, (c–e) 5 wt%, 8 wt%, and 10 wt% WO_3_/TiO_2_; (B) hydrothermally prepared (a) hyd-TiO_2_, (b) hyd-WO_3_, (c) TiO_2_@WO_3_ and (d) WO_3_@TiO_2_.

The XRD patterns for the obtained nanocomposites are shown in Fig. 1. The sol-TiO_2_ showed peaks at 2*θ* = 25.4°, 38.0°, 48.1°, 54.1°, 55.2°, 62.8°, and 75.3°, which correspond to the (101), (004), (200), (105), (211), (204), and (215) planes of the hexagonal crystal lattice of the TiO_2_ anatase phase (JCPDS PDF 89-4921), while the sole diffraction peak at 31.0° (marked by an asterisk in Fig. 1A(a)) corresponds to the (121) plane of the orthorhombic crystal lattice of brookite phase of TiO_2_ (JCPDS PDF 29-1360).

The sol-WO_3_ showed peaks at 2*θ* = 14.2°, 23.1°, 24.5°, 28.4°, 33.8°, 36.8°, 50.0°, 52.3°, 55.6°, 58.2°, and 63.5°, which were, respectively assigned to the (100), (002), (110), (200), (112), (202), (220), (213), (222), (312), and (402) planes of the hexagonal crystal lattice of WO_3_ (JCPDS PDF 85-2459). In the 5 wt% WO_3_/TiO_2_ nanocomposite, the presence of WO_3_ was not well confirmed, which was probably due to the low concentration of WO_3_.

The 8 wt% and 10 wt% WO_3_/TiO_2_ nanocomposites showed diffraction peaks for WO_3_; however, some peaks (marked by ♦) significantly shifted from those of sol-WO_3_ and were identified as a monoclinic phase of the tungsten oxide W_18_O_49_ (JCPDS PDF 05-0392). In contrast, the XRD peaks for TiO_2_ in these nanocomposites appeared at the same positions as those in sol-TiO_2_. These results suggested that the W ions were minor components in the nanocomposites (only 8 wt% and 10 wt%) and formed new crystal structures under the influence of Ti compounds.

The hyd-TiO_2_ exhibited diffraction peaks at 2*θ* = 25.4°, 38.0°, 48.1°, 54.1°, 55.2°, 62.8°, and 75.2°, which correspond to the (101), (112), (200), (105), (211), (204), and (215) planes, respectively (Fig. 1B(a)). All the observed diffraction peaks belonged to anatase TiO_2_ (JCPDS PDF 89-4921), and the peak for the (121) plane of the brookite phase was not observed. The hyd-WO_3_ showed diffraction patterns at 2*θ* = 23.4°, 23.9°, 24.6°, 26.8°, 28.9°, 33.5°, 34.4°, 35.7°, 42.1°, 47.5°, 48.5°, 50.1°, 53.6°, 56.1°, 62.4°, and 76.6° corresponding to the (001), (020), (200), (1̄20), (1̄11), (2̄01), (220), (121), (221), (002), (040), (140), (022), (4̄20), (3̄40), and (422) planes, respectively, which indicated a monoclinic WO_3_ crystal (JCPDS PDF 05-0363). Thus, in contrast with the sol–gel method, the hydrothermal synthesis process could be adjusted to result in the monoclinic crystalline phase. Both the hydrothermally produced core–shell TiO_2_@WO_3_ and WO_3_@TiO_2_ showed diffraction peaks for anatase TiO_2_, and peaks for WO_3_ were not observed. The weakness of the XRD peak intensity of WO_3_ suggested that the WO_3_ shell was very thin and that the WO_3_ core was covered with a thick TiO_2_ shell.

The crystallite size (*D*) was computed from Debye–Scherrer's equation (eqn (5)).^52^5
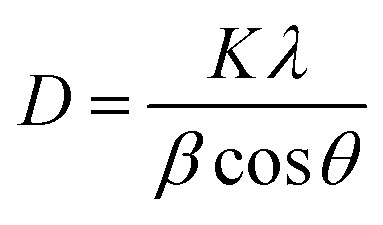
where *K* is the Scherrer constant (0.9), λ is the wavelength of the X-ray for CuKα (1.54184 Å), *β* is the FWHM of the peak in radians, and *θ* is the diffraction angle in radians. The estimated average crystallite sizes of sol-TiO_2_ (from peaks at 2*θ* = 25.4°, 31.0°, 38.0°, 48.1°, 54.1°, 55.1°, and 62.8°), sol-WO_3_ (using all observed peaks), 5 wt% (from peaks at 2*θ* = 25.5°, 38.1°, 48.2°, 54.3°, and 62.9°), 8 wt% (from peaks at 2*θ* = 16.9°, 25.4°, 27.7°, 32.6°, 38.0°, and 48.0°), and 10 wt% WO_3_/TiO_2_ (from peaks at 2*θ* = 16.9°, 25.5°, 27.7°, 32.6°, 38.0°, and 48.1°) nanoparticles were 7.9 nm, 13.6 nm, 8.7 nm (for TiO_2_), 14.7 nm (for TiO_2_), and 20.1 nm (for TiO_2_), respectively. For the hydrothermally synthesized materials, the observed average sizes of hyd-TiO_2_ (from peaks at 2*θ* = 25.4°, 38.0°, 48.1°, 54.1°, 55.2°, and 62.8°), hyd-WO_3_ (using all diffraction peaks), TiO_2_@WO_3_ (from peaks at 2*θ* = 25.5°, 38.0°, 48.3°, 54.2°, 55.3°, and 62.9°), and WO_3_@TiO_2_ (from peaks at 2*θ* = 25.5°, 38.0°, 48.2°, 54.2°, 55.3°, and 62.8°) were 9.4 nm, 24.1 nm, 9.6 nm (for TiO_2_), and 7.4 nm (for TiO_2_), respectively. These results demonstrated that the preparation methods and the mixing ratios altered the crystallite sizes. The hydrothermal process could lead to larger crystals in TiO_2_ and WO_3_ than the sol–gel process, and a higher content of tungsten could result in larger crystals of TiO_2_.

### 3.2 Optical properties of nanocomposites

**Fig. 2:**
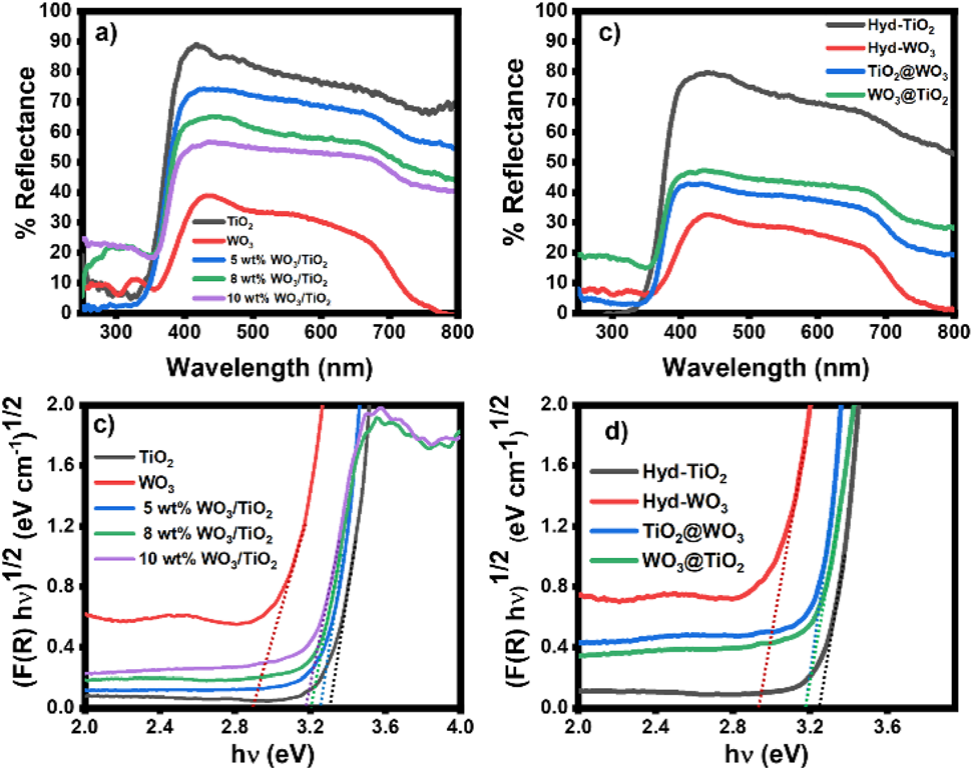
(a and b) UV-VIS DRS spectra of the sol–gel and hydrothermally prepared samples and (c and d) bandgap energy analyses of nanocomposites.

The optical properties of the nanomaterials were studied with UV-VIS DRS (Fig. 2). Both sol-TiO_2_ and hyd-TiO_2_ exhibited high reflectance in the range greater than 350 nm, and with the addition of WO_3_, the material reflectance decreased in this range (Fig. 2a and b). The reflectance of TiO_2_@WO_3_ was slightly lower than that of WO_3_@TiO_2_. Since WO_3_ can absorb light of wavelengths shorter than 500 nm,^31,32,56^ the lower reflectance could be partially explained by the photoexcitation of WO_3_ in the short wavelength region. At longer wavelengths (>500 nm), the WO_3_ and WO_3_–TiO_2_ nanocomposites showed lower reflectance than TiO_2_. This could be explained by the existence of WO_x_ (2 < *x* < 3), which could absorb light, and by the structures of the film specimens.^32^

The bandgap energy (*E*_g_) of the metal oxides was determined using the Kubelka–Munk function and Tauc plots (Fig. 2c and d).^57^ The *E*_g_ values were evaluated from the intercepts of the energy axis. The *E*_g_ values of TiO_2_ and WO_3_ were 3.2–3.3 eV and 2.8–2.9 eV, respectively, and compared with the materials prepared by the sol–gel method, hyd-TiO_2_ and hyd-WO_3_ showed slightly decreased *E*_g_ values, which was attributed to their larger crystal sizes.^58^ The nanocomposites had *E*_g_ values between those of TiO_2_ and WO_3_: the sol–gel method resulted in *E*_g_ values of 3.25, 3.20, and 3.18 eV, corresponding to 5 wt%, 8 wt%, and 10 wt% WO_3_/TiO_2_, respectively (Fig. 2c). For core–shell TiO_2_@WO_3_ and WO_3_@TiO_2_, the *E*_g_ values were nearly identical at 3.18 eV (Fig. 2d). The intermediate *E*_g_ values (in between those of TiO_2_ and WO_3_) suggested interactions between TiO_2_ and WO_3_ in both the mixed and core–shell structures. Mutual effects in the nanocomposites can decrease the electron–hole recombination rate at the interface for TiO_2_ and WO_3_.

### 3.3 Morphological analyses of nanocomposites

**Fig. 3:**
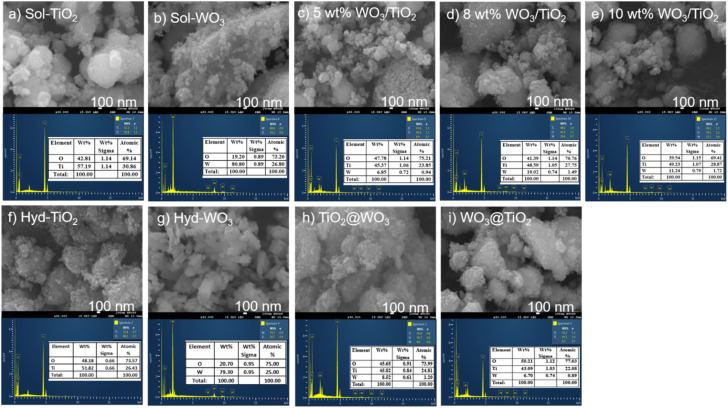
SEM images (upper) and EDX (lower) results for sol–gel (a–e) and hydrothermally (f–i) synthesized materials.

The morphologies of the nanocomposites were observed using SEM (Fig. 3). The materials prepared by the sol–gel method consisted of aggregated spherical nanoparticles, and no systematic difference was observed. The hyd-TiO_2_ consisted of aggregates of small nanoparticles, while the hyd-WO_3_ showed large crystalline structures, as indicated by the XRD analysis. However, the surface morphology of the core–shell nanocomposites did not show a significant difference. This suggests that the WO_3_ shell in the TiO_2_@WO_3_ nanocomposite could not develop large crystals because it grew from small TiO_2_ particles.

The EDX results identified the presence of titanium (Ti) and oxygen (O) in TiO_2_, tungsten (W) and O in the WO_3_ oxide, as well as the presence of Ti, W, and O in the nanocomposites obtained from both the sol–gel and hydrothermal methods. The weight ratios of W to Ti in the sol–gel and core–shell nanocomposites are shown in Table 1. The experimentally obtained W ratios in the nanocomposites prepared by the sol–gel method were significantly higher than the corresponding theoretical values, which suggests that a significant amount of Ti compounds were not recovered during the preparation process relative to the W compounds. In contrast, the hydrothermally prepared nanocomposites showed W ratios that agreed well with the theoretical values. This suggests that the hydrothermal process immobilized the Ti compounds in the nanocomposites during crystal growth. The higher W ratio in TiO_2_@WO_3_ relative to WO_3_@TiO_2_ could be explained by the fact that the TiO_2_ core was covered by the WO_3_ shell.

**Table 1:** Weight ratios of elements in the nanocomposites as analyzed by the EDX and XPS methods

Nanocomposite	Synthesis method	EDS result	XPS result	Theoretical ratio
TiO_2_ (Ti : O)	Sol–gel process	1.3		1.4
WO_3_ (W : O)		4.2		3.8
5 wt% WO_3_/TiO_2_ (W : Ti)		0.15	0.22	0.07
8 wt% WO_3_/TiO_2_ (W : Ti)		0.21	0.28	0.12
10 wt% WO_3_/TiO_2_ (W : Ti)		0.23	0.33	0.15
TiO_2_ (Ti : O)	Hydrothermal process	1.1		1.4
WO_3_ (W : O)		3.8		3.8
TiO_2_@WO_3_ (W : Ti)		0.18	0.26	0.15
WO_3_@TiO_2_ (W : Ti)		0.16	0.20	0.15

### 3.4 Chemical states of nanocomposites

**Fig. 4:**
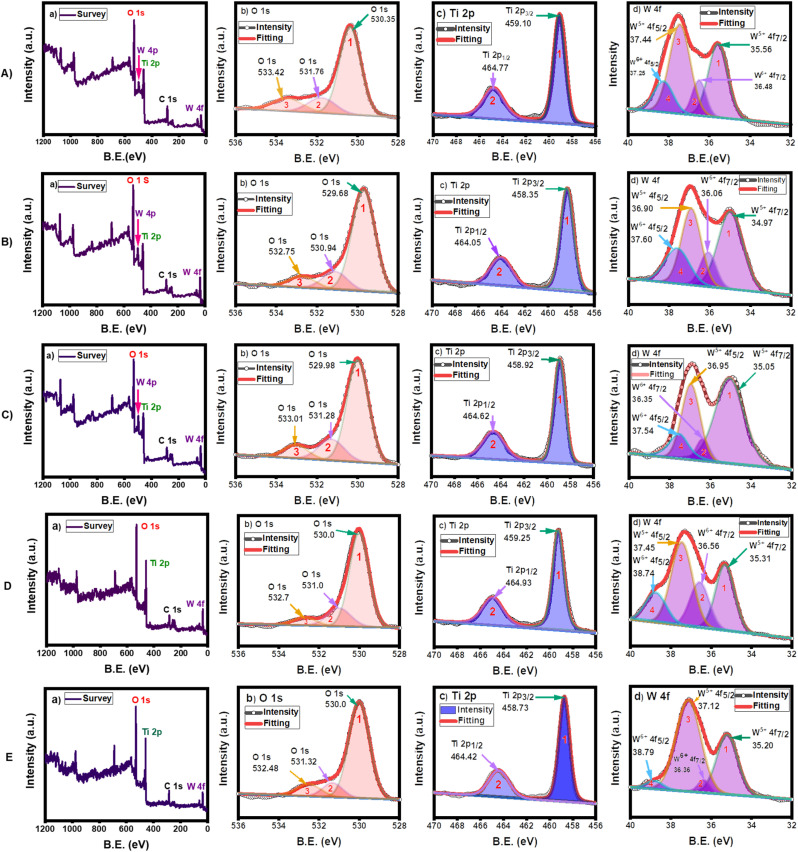
XPS survey and high-resolution spectra of the sol–gel method: (A) 5 wt% WO_3_/TiO_2_, (B) 8 wt% WO_3_/TiO_2_ and (C) 10 wt% WO_3_/TiO_2_, and core–shell method: (D) TiO_2_@WO_3_ and (E) WO_3_@TiO_2_ with (a) survey, (b) O 1s, (c) Ti 2p, and (d) W 4f.

XPS analysis was conducted to determine the chemical states of the sol–gel synthesized and hydrothermally synthesized nanocomposites (Fig. 4). The peak parameters are shown in the ESI (Tables S1–S3 in the ESI†). The survey XPS spectrum showed the existence of oxygen (O 1s), titanium (Ti 2p), tungsten (W 4p and 4f), and carbon (C 1s) on the material surfaces.^44^ The carbon contribution originated from the substrate and was used to calibrate the binding energy.

The deconvoluted XPS spectra of O 1s showed three distinct peaks in all specimens. The peak at the lowest binding energy (530.4 eV for 5 wt%, 529.7 eV for 8 wt%, 530.0 eV for 10 wt% WO_3_/TiO_2_, 530.0 eV for TiO_2_@WO_3_, and 530.0 eV for WO_3_@TiO_2_) was attributed to the lattice oxygen (O^2−^) of O–Ti bonds in TiO_2_ and O–W bonds in WO_3_,^40^ whereas the middle peaks (531.8 eV for 5 wt%, 530.9 eV for 8 wt%, 531.3 eV for 10 wt% WO_3_/TiO_2_, 531.0 eV for TiO_2_@WO_3_, and 531.3 eV for WO_3_@TiO_2_) could be attributed to substoichiometric WO_x_ (2 < *x* < 3)^40,59,60^ or hydroxide groups adsorbed on the oxide surface as W–O–H and Ti–O–H,^40^ which correspond to oxygen vacancies. To compensate for the charge imbalance in the oxygen-deficient state, OH groups were bound to the metal cations. Thus, the density of oxygen vacancies is indicated by the intensity of these mid-binding energy peaks.^61^ The third peaks, which were located at the highest binding energy (533.4 eV for 5 wt%, 532.8 eV for 8 wt%, 533.0 eV for 10 wt% WO_3_/TiO_2_, 532.7 eV for TiO_2_@WO_3_, and 532.5 eV for WO_3_@TiO_2_), could be attributed to contamination from oxygen-containing hydrocarbons,^40^ H_2_O,^59^ or surface-adsorbed O_2_.^62^ The peak areas (%) of the mid-binding energy peaks ranged from 11% to 18%, which indicated that significant numbers of oxygen vacancies were formed in the nanocomposites, as suggested from the UV-VIS DRS spectra (Fig. 2). These oxygen vacancies could extend the lifetime of the charge carriers and increase the photocatalytic activity of these catalysts.

The presence of only one Ti 2p doublet for Ti 2p_3/2_ and Ti 2p_1/2_ indicated that all Ti atoms shared the same oxidation state (Ti^4+^).^40^ The binding energies of Ti 2p_3/2_ for the sol–gel synthesized nanocomposites of 5 wt%, 8 wt%, and 10 wt% WO_3_/TiO_2_ were 459.1 eV, 458.4 eV, and 458.9 eV, respectively, while they were 459.3 eV and 458.7 eV for the hydrothermally prepared TiO_2_@WO_3_ and WO_3_@TiO_2,_ respectively. The binding energies of Ti 2p_1/2_ for 5 wt%, 8 wt%, and 10 wt% WO_3_/TiO_2_ were 464.8 eV, 464.0 eV, and 464.6 eV, respectively, whereas they were 464.9 eV and 464.4 eV for TiO_2_@WO_3_ and WO_3_@TiO_2,_ respectively. The minor change in energy in the nanocomposites could be attributed to the interactions of W–O–Ti bonds; however, the changes were not significantly or systematic.

The peaks of W 4f appeared as two doublets. The first pair (peaks 1 and 3) might have arisen from W^5+^ in substoichiometric WO_*x*_ (2 < *x* < 3),^40^ which corresponds to an oxygen vacancy. The second pair (peaks 2 and 4) was ascribed to W^6+^ in WO_3_.^61^ The ratio of W 4f to Ti 2p determined from the peak area in the nanocomposites (Tables 1 and S3 in the ESI†) was 0.22, 0.28, 0.33, 0.26, and 0.20 for 5 wt%, 8 wt%, 10 wt% WO_3_/TiO_2_, TiO_2_@WO_3_, and WO_3_@TiO_2,_ respectively. These values were larger than those obtained by the EDX method, which suggested that the W component existed more on the surface than in the bulk phase of the nanocomposites. The detection of W in the core–shell WO_3_@TiO_2_ nanocomposite and Ti in the core–shell TiO_2_@WO_3_ nanocomposite suggested that the core–shell structures were imperfect, although the compositions were controlled to an extent.

**Fig. 5:**
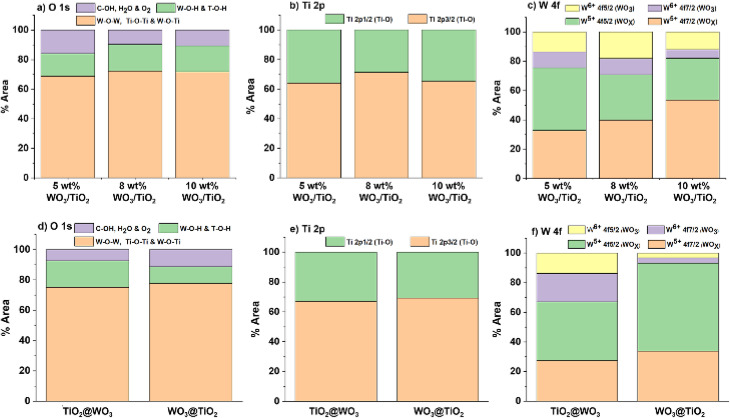
Ratios of chemical states for nanocomposites prepared by the sol–gel method ((a) O 1s, (b) Ti 2p, and (c) W 4f) and the core–shell method ((d) O 1s, (e) Ti 2p, and (f) W 4f).

Fig. 5 shows the ratios of the %area of XPS peaks for each element in the nanocomposites. The changes in the %area of both W^5+^ and W^6+^ in the nanocomposites indicated the high ratios of W^5+^ on the surface of the nanocomposites. The existence of W^5+^ can extend the light absorption range, and W^5+^ can provide an electron to molecular oxygen to form superoxide radicals (O_2_˙^−^) under light irradiation. Therefore, a higher ratio of W^5+^ on the surface could be an advantage for photocatalysis. On the other hand, the low positive charge on the surface induces low attractive interactions with negatively charged dyes, which is disadvantageous for photocatalysis.

### 3.5 Adsorption process of dyes

The adsorption behaviors of cationic MB^+^ and anionic MO^−^ onto the nanocomposites were investigated in the range of 1–8 mg L^−1^ initial dye concentrations (the data and the fitting curves are shown in Fig. S2 and S3 in the ESI†). The adsorption performances of the pure oxides and composite materials were evaluated by the Langmuir isotherm adsorption models.^63–65^ The Langmuir adsorption isotherms of the dyes are depicted in eqn (6) below:6
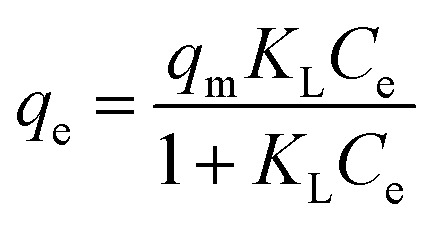
where *q*_e_ is the equilibrium adsorption capacity (mg g^−1^) at a specific dye concentration, *q*_m_ is the adsorption maximum capacity of adsorbents (mg g^−1^) when the concentration of dye is sufficiently high, *K*_L_ is the Langmuir adsorption constant (L mol^−1^), and *C*_e_ is the equilibrium free dye concentration (mg L^−1^). The experimental values of *q*_m_ and *K*_L_ were fitted with the nonlinear fitted Langmuir isotherm adsorption curves. The fitting equations are given in Table 2.

**Table 2:** The Langmuir adsorption model parameters for the MB^+^ and MO^−^ adsorption process and the Gibbs free energy change in the adsorption process

	Nanocomposite	*q*_m_ (mg g^−1^)	*K*_L_ (×10^5^ L mol^−1^)	*R* ^2^	Δ*G* (K J mol^−1^)
MB^+^	Sol-TiO_2_	6.6	3.64	0.9038	−31.7
	hyd-TiO_2_	13.1	2.76	0.9887	−31.0
	Sol-WO_3_	31.2	31.82	0.9679	−37.1
	hyd-WO_3_	35.6	27.91	0.9279	−36.8
	5 wt% WO_3_/TiO_2_	38.7	5.55	0.9798	−32.9
	8 wt% WO_3_/TiO_2_	40.8	6.41	0.9684	−33.1
	10 wt% WO_3_/TiO_2_	38.5	7.63	0.9768	−33.6
	TiO_2_@WO_3_	55.8	5.94	0.9533	−33.0
	WO_3_@TiO_2_	39.0	4.34	0.9643	−32.2
MO^−^	Sol-TiO_2_	3.04	2.67	0.349	−31.0
	hyd-TiO_2_	1.62	2.85	0.7081	−31.1
	Sol-WO_3_	0.24	0.77	0.7724	−27.9
	hyd-WO_3_	0.23	0.79	0.7303	−27.9
	5 wt% WO_3_/TiO_2_	0.55	2.07	0.6907	−30.3
	8 wt% WO_3_/TiO_2_	0.39	1.09	0.8428	−28.7
	10 wt% WO_3_/TiO_2_	0.46	0.82	0.6463	−28.0
	TiO_2_@WO_3_	0.27	1.21	0.6739	−29.0
	WO_3_@TiO_2_	0.73	2.34	0.5965	−30.6

**Fig. 6:**
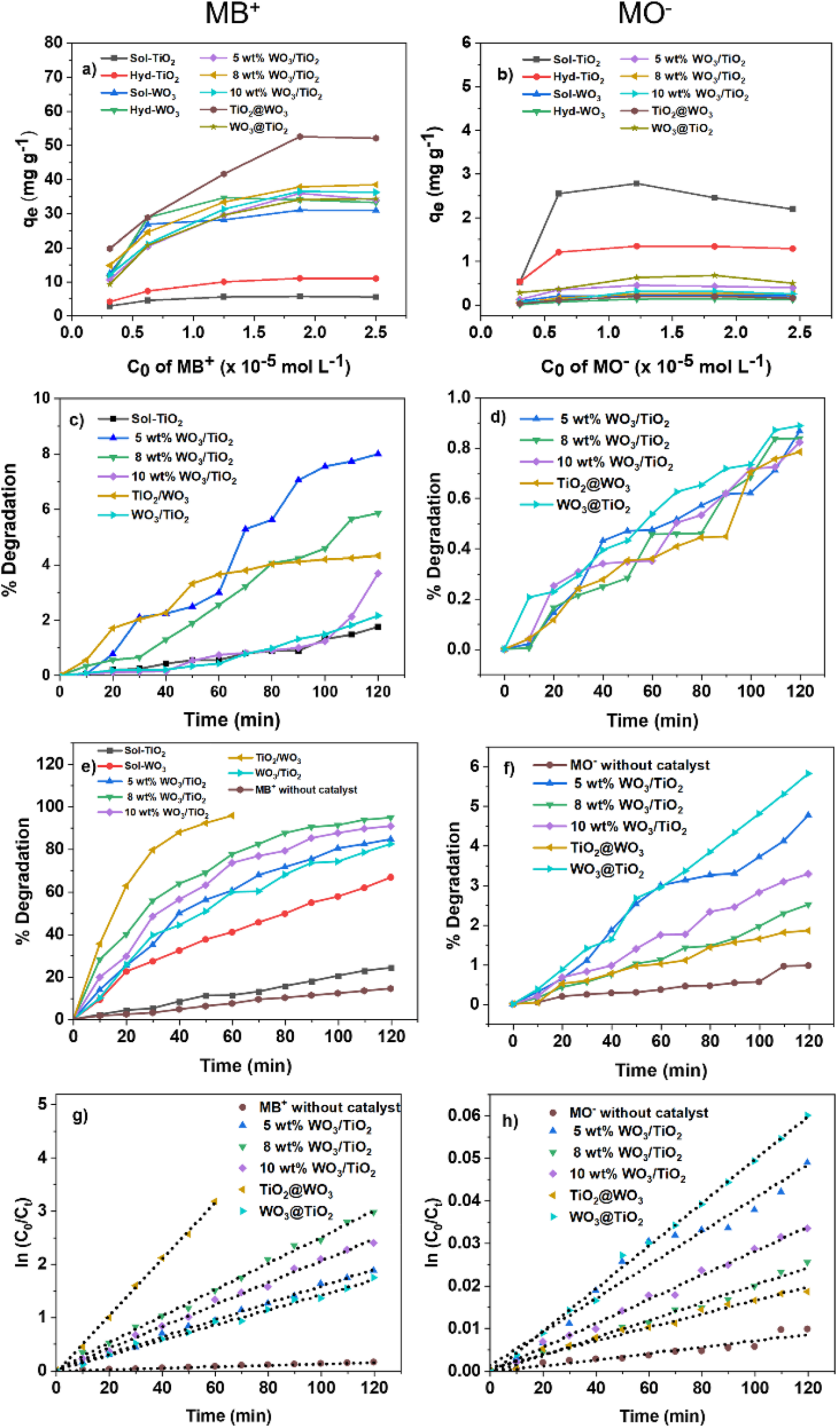
(a & b) Adsorbed amounts of MB^+^ and MO^−^ on TiO_2_, WO_3_, and nanocomposites at various concentrations, respectively; (c &d) MB^+^ and MO^−^ decomposition in dark; (e &f) degradation rates for MB^+^ and MO^−^ under UV light; (g & h) pseudo-first-order kinetic model for decomposition of MB^+^ and MO^−^ under UV light. Left column is for MB^+^ and right column is for MO^−^, respectively.

The adsorption of MB^+^ from different initial concentrations was explored at pH 7.4 without pH control. The adsorption of different MO^−^ initial concentrations was probed at pH 6.7 without pH control. As observed in Fig. 6a, the amount of adsorbed MB^+^ (*q*_e_) rose as the initial concentration increased and became saturated at high concentrations (6–8 ppm). The amount of adsorbed MO^−^ (*q*_e_) also increased as the initial concentration increased in the low concentration range (1–2 ppm) and became almost saturated at higher concentrations (2–8 ppm), except for a slight increase and decline for TiO_2_ (Fig. 6b). For the adsorption of MB^+^, the Langmuir model fitted well (*R*^2^ > 0.9), indicating that the adsorption of MB^+^ on the nanocomposites followed a monolayer adsorption process. However, the Langmuir model did not yield good fits for adsorption of MO^−^ (*R*^2^ was in the range of 0.3490 to 0.8428), which could be explained by the low adsorption amounts of MO^−^ (two orders of magnitude smaller than those of MB^+^, except for TiO_2_), leading to large errors.

Then, dye adsorption on the nanocomposites was analyzed with eqn (7).^5^

7 Δ*G* = −*RT* ln *K*_L_

where Δ*G* is the Gibbs free energy change, *R* is the gas constant (8.314 J mol^−1^ K^−1^), and *T* is the absolute temperature (298 K). The large Δ*G* of MB^+^ adsorption could be due to the electrostatic interactions between the metal oxides (negatively charged) and the cationic dye (Table 2). In general, *K*_L_ and Δ*G* increased as the tungsten component increased, which suggested that WO_3_ promoted dye adsorption. According to the reported zeta potential measurements of TiO_2_, the surface charge of TiO_2_ is positive over the pH range of 2.50 to 7.35,^66^ and therefore, the surface of TiO_2_ in this study (pH 7.4) was slightly negatively charged. In contrast, the neutral point of WO_3_ is pH 1.9,^67^ and therefore, a higher proportion of tungsten resulted in a more negative charge on the surface. The difference in the *K*_L_ and the Δ*G* of the core–shell TiO_2_@WO_3_ and WO_3_@TiO_2_ nanocomposites demonstrated the enhanced adsorption of MB^+^ on WO_3_. However, the nanocomposites provided a higher adsorption capacity than WO_3_, despite their lower interactions with MB^+^: this could be explained by the large surface area of the nanocomposites. A comparison of preparation methods shows that sol-TiO_2_ and sol-WO_3_ resulted in stronger interactions (higher *K*_L_ and Δ*G*) but lower adsorption capacities than hyd-TiO_2_ and hyd-WO_3_, respectively. As the XRD analysis indicated, the sol–gel method provided smaller crystal grains; however, these crystals were aggregated, and the surface area available for adsorption was likely to be limited (Fig. 1 and 3). The stronger interaction of the sol-metal oxides with MB^+^ could be due to the defects on the surface of the sol-metal oxides, which served as pockets. Among the nanocomposites, the hydrothermally prepared TiO_2_@WO_3_ provided a higher adsorption capacity, although the *K*_L_ and Δ*G* were similar to those for the 5 wt% WO_3_/TiO_2_ nanocomposite, which had a lower W-component on its surface (Table 1). Therefore, in terms of availability and efficiency, the W component was more effectively utilized for adsorbing MB^+^ in the nanocomposites prepared by the sol–gel method than those prepared by the hydrothermal method. The advantage of TiO_2_@WO_3_ was its large surface area, which allowed for a high adsorption capacity, and this was achieved by the secondary deposition of WO_3_.

In terms of the adsorption behavior of MO^−^, it should be noted that the Δ*G* of MO^−^ adsorption could not be precisely estimated because of the poor correlation coefficients (<0.9) obtained. However, TiO_2_ showed a higher adsorption capacity than the others for MO^−^ adsorption, and the nanocomposites also exhibited stronger interactions due to the higher compositional ratio of titanium, as expected from the surface charge of the nanocomposites. The *K*_L_ and Δ*G* of TiO_2_ for MO^−^ were comparable to those for MB^+^ adsorption. This finding suggests that the TiO_2_ nanoparticles provided binding sites for both the cationic and anionic dyes. The lower adsorption capacity of hyd-TiO_2_ for MO^−^ compared with MB^+^ suggests that the number of cationic binding sites was lower in hyd-TiO_2_, which could be due to the difference in crystallinity and the crystal structures shown by the XRD measurements (Fig. 1). The tungsten enhanced the adsorption of MB^+^ and weakened the adsorption of MO^−^ on the nanocomposite surfaces.^68^ However, the *K*_L_ and Δ*G* values indicated that some nanocomposites also provided effective binding sites for MO^−^, especially the 5 wt% WO_3_/TiO_2_ and WO_3_@TiO_2_ nanocomposites.

### 3.6 Photocatalysis of dyes

The photocatalytic activity of the synthesized materials was evaluated by dye decomposition. The resulting absorption spectra are shown in Fig. S4 and S5 in the ESI.† The decomposition curves and their kinetic analyses for MB^+^ and MO^−^ are shown in Fig. 6.

The MB^+^ was irradiated with UV light in an aqueous solution of pH 7.4. Measurements were also performed under dark conditions, and the decrease in concentration was in the range of 1–8% of the initial concentration, which could have been caused by the disaggregation of the nanocomposites induced by stirring. In the absence of nanocomposite materials, the degradation of MB^+^ was 14.4% under UV irradiation, which was lower than that in the presence of nanocomposites. Compared with TiO_2_ and WO_3_, the nanocomposites exhibited better photocatalytic activity under UV light irradiation. The photocatalytic activity of 8 wt% WO_3_/TiO_2_ was highest among the sol–gel nanocomposites, with 94.9% decomposition after 2 h, while 5 wt% WO_3_/TiO_2_ achieved the lowest degradation (84.7%) during the same irradiation time. The core–shell TiO_2_@WO_3_ demonstrated superior photocatalytic activity, with 95.8% decomposition after 1 h of UV irradiation, while WO_3_@TiO_2_ demonstrated lower activity (decomposition of 82.5%) after 2 h than 5 wt% TiO_2_@WO_3_. The reaction rates were analyzed as pseudo first-order reactions using eqn (4) and the Langmuir–Hinshelwood model.^65^ The rate constant 
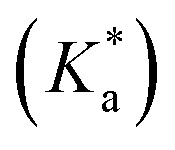
 for each nanocomposite and dye is summarized in Table 3.

**Table 3:** Rate constants of dye decomposition as pseudo first-order reactions for MB^+^ and MO^−^

Photocatalysts	MB^+^			MO^−^		
	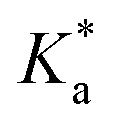 (×10^−2^ min^−1^)	*R* ^2^	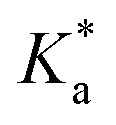 per (×10^−5^*K*_L_ × *q*_m_)	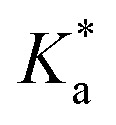 (×10^−2^ min^−1^)	*R* ^2^	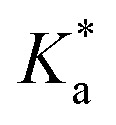 per (×10^−5^*K*_L_ × *q*_m_)
Sol-TiO_2_	0.23	0.9904	9.57	0.1	0.9816	12.32
Sol-WO_3_	0.86	0.9927	0.87	0.01	0.9335	54.11
5 wt% WO_3_/TiO_2_	1.59	0.9974	7.40	0.04	0.9634	35.12
8 wt% WO_3_/TiO_2_	2.48	0.9967	9.48	0.02	0.9905	47.05
10 wt% WO_3_/TiO_2_	2.05	0.9959	6.98	0.03	0.9930	79.53
Hyd-TiO_2_	0.46	0.9978	12.72	0.07	0.9605	15.16
Hyd-WO_3_	1.04	0.9925	1.05	0.008	0.9409	44.03
TiO_2_@WO_3_	5.33	0.9986	16.08	0.02	0.9778	61.22
WO_3_@TiO_2_	1.41	0.9930	8.33	0.05	0.9962	29.27
Without catalyst	0.13	0.9954	—	0.007	0.9067	—

These activities were inconsistent with the orders of both the adsorption rate (*K*_L_) and the adsorption capacity (*q*_m_) of the nanocomposites (Table 2). To some extent, the magnitude of the reaction rates could be explained by several factors: (1) the tungsten in the nanocomposites provided higher reaction rates due to its strong interaction with MB^+^, (2) the absorbance of TiO_2_ was low at an excitation wavelength of 365 nm, (3) among the sol–gel nanocomposites, the adsorption capacities determined the order of the reaction rates, and (4) TiO_2_@WO_3_ showed highest rate constant, which could because it also had the highest adsorption capacity. However, these explanations were not sufficient for explaining the lower rate constant of WO_3_, which had a high adsorption constant and moderate adsorption capacity. Upon dividing 
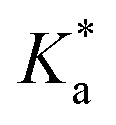
 by *K*_L_ and *q*_m_, WO_3_ exhibited the lowest rate constant per (adsorption rate x adsorption mass), while that of TiO_2_@WO_3_ was the highest, followed by that of hyd-TiO_2_. Therefore, the photocatalytic activity was not determined only by the adsorption amount and the adsorption rate.

For comparison, the anionic MO^−^ was also degraded at pH 6.7 under UV light irradiation. The reaction rate was much lower than that of MB^+^, which could be expected from the adsorption parameters discussed above (Table 2). Among the nanocomposites, WO_3_@TiO_2_ demonstrated the highest efficiency (degradation of ∼6% MO^−^), whereas TiO_2_@WO_3_ demonstrated the lowest efficiency (degradation of ∼2% MO^−^). The negative surface charge from the tungsten oxides had adverse effects on the photocatalysis of MO^−^. Upon dividing 
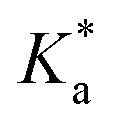
 by *K*_L_ and *q*_m_, the nanocomposites demonstrated a clear tendency: a higher composition of tungsten resulted in a higher rate constant. This could be explained by the stronger light absorption at 365 nm by tungsten components. However, the higher rate constants per (adsorption rate × adsorption mass) of 10 wt% WO_3_/TiO_2_ and TiO_2_@WO_3_ suggested that the nanocomposite decomposed MO^−^ more effectively than TiO_2_ and WO_3_.

Compared with other studies for the MB^+^ degradation performance (Table S4 in the ESI†), the activity of 8 wt% WO_3_/TiO_2_ was 1.34 times higher than that of 25 wt% mixed WO_3_/TiO_2_.^46^ The activity of the core–shell TiO_2_@WO_3_ of the current study was 3.59 times higher than that of 36 wt% core–shell WO_3_/TiO_2_.^43^ These results indicate that the large amount of WO_3_ is not essential for the effective photocatalyst.

### 3.7 Dye decomposition mechanism

**Fig. 7:**
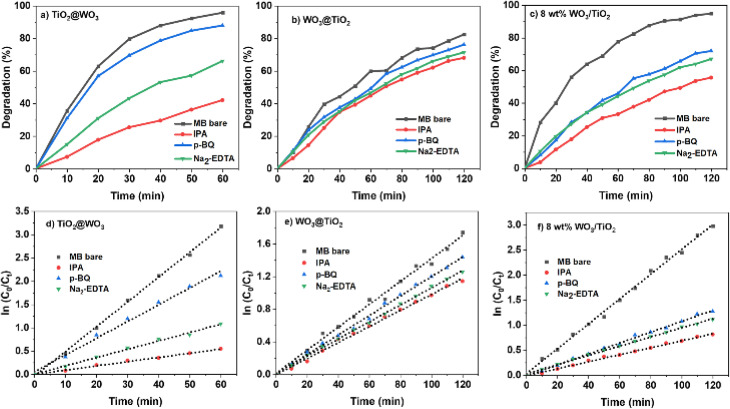
(a–c) Photocatalytic decomposition of MB^+^ over core–shell TiO_2_@WO_3,_ WO_3_@TiO_2,_ and coprecipitated 8 wt% WO_3_/TiO_2_ with various scavengers under UV light irradiation; (d–f) kinetic analysis of MB^+^ decomposition.

To study the active species present during photocatalysis with the nanocomposites, dye photocatalysis was analyzed in the presence of active species scavengers. The active species generated by TiO_2_ and WO_3_ are considered to be superoxide anions (O_2_^−^), holes (h^+^), and hydroxyl radicals (HO˙), which can be scavenged by *p*-BQ, Na_2_-EDTA, and IPA, respectively.^38,48,69,70^ The degradation curves and the kinetic analyses are shown in Fig. 7 and Table 4.

**Table 4:** Effect of active species scavengers on the MB decomposition efficiency (%*D*)

Sample type	8 wt% WO_3_/TiO_2_				TiO_2_@WO_3_				WO_3_@TiO_2_			
	%*D*	*K*_a_ (×10^−2^ min^−1^)	*R* ^2^	Inhibition	%*D*	*K*_a_ (×10^−2^ min^−1^)	*R* ^2^	Inhibition	%*D*	*K*_a_ (×10^−2^ min^−1^)	*R* ^2^	Inhibition
Without scavenger	94.9	2.48	0.9967	—	95.8	5.33	0.9986	—	82.5	1.41	0.993	—
*p*-BQ	72.0	1.09	0.9969	−56%	87.9	3.60	0.9931	−32%	76.2	1.20	0.9974	−15%
Na_2_-EDTA	67.0	0.92	0.9981	−63%	66.1	1.79	0.9941	−66%	71.5	1.05	0.9992	−26%
IPA	55.6	0.70	0.9969	−72%	42.1	0.91	0.9955	−83%	68.1	0.99	0.9968	−30%

The addition of scavengers decreased the decomposition rate in all cases. These results indicated that hydroxyl radicals were the most active species for all the photocatalysts examined. When IPA was used to quench hydroxyl radicals, the *K*_a_ values of the photocatalysts decreased to a similar level (0.70–0.99 × 10^−2^ min^−1^). The activity of TiO_2_@WO_3_ exhibited the greatest decrease with the use of IPA, and the effect of IPA on WO_3_@TiO_2_ was the smallest. This suggests that WO_3_ on the nanocomposite surface mainly provides hydroxyl radicals as the active species; the OH^−^ groups attached to W^5+/6+^ could be directly oxidized by holes to generate hydroxyl radicals.

A comparison of the effects of *p*-BQ and Na_2_-EDTA show that the *K*_a_ of TiO_2_@WO_3_ decreased more with the addition of Na_2_-EDTA than *p*-BQ, while the 8 wt% WO_3_/TiO_2_ nanocomposite was similarly quenched by both scavengers. These results suggest that the activity of the core–shell TiO_2_@WO_3_ depended on the activity of holes more than photoexcited electrons, while the codeposited WO_3_/TiO_2_ used both to a similar extent. In the reversed structure, the core–shell WO_3_@TiO_2_ nanocomposite also demonstrated a stronger effect with Na_2_-EDTA than *p*-BQ; however, its activity was much lower than that of TiO_2_@WO_3,_ and the decrease in the *K*_a_ value was not large. The effects of Na_2_-EDTA and *p*-BQ (*i.e.*, the amounts of scavenged holes and electrons) were not greatly different for WO_3_@TiO_2_, and the *K*_a_ values became similar to those of 8 wt% WO_3_/TiO_2_. The weak effects of scavengers on WO_3_@TiO_2_ suggested that the active species (O_2_^−^, hole, and HO˙) were rapidly changed to other species or quenched in the nanocomposite by recombination.

**Fig. 8:**
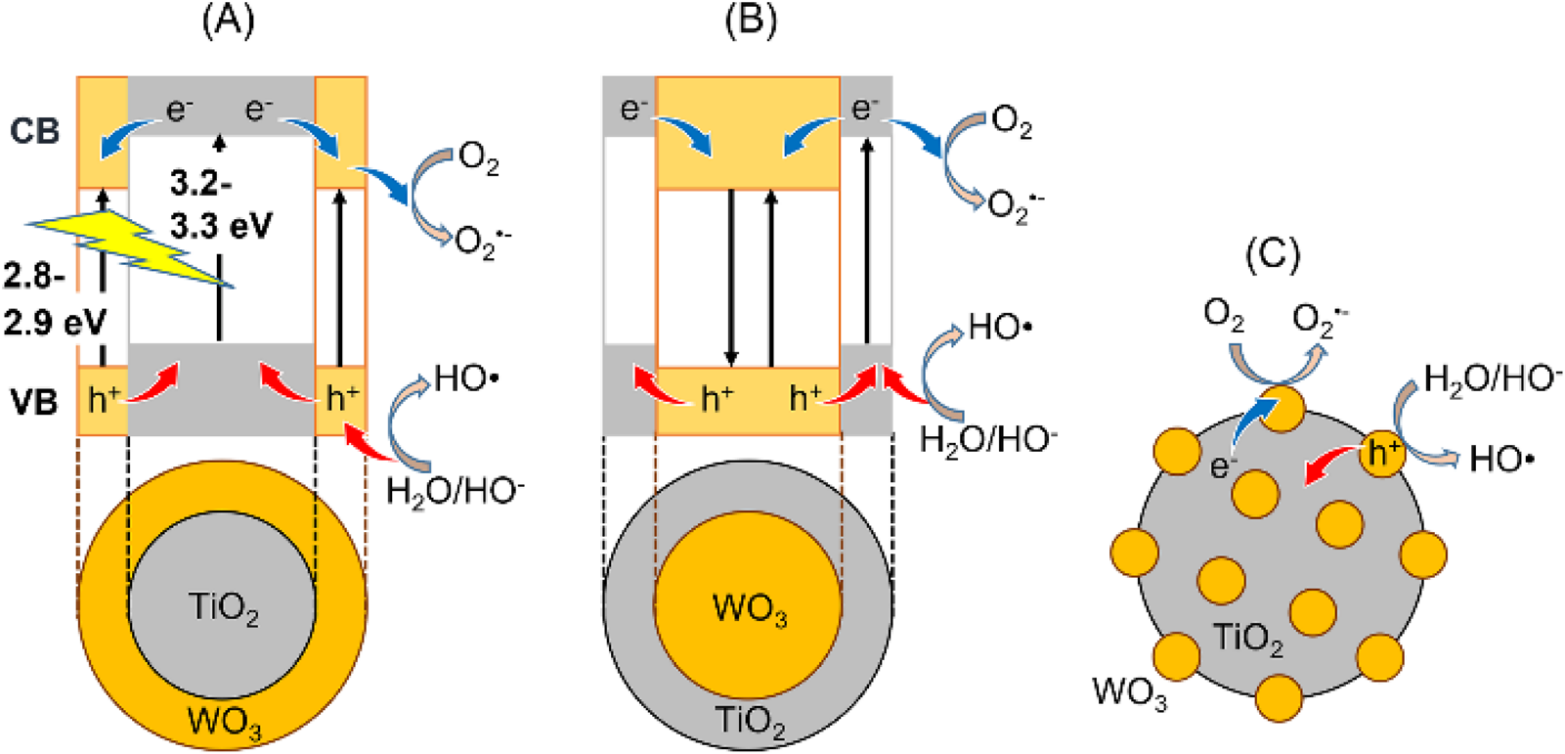
Dye decomposition mechanism using (A) TiO_2_@WO_3_, (B) WO_3_@TiO_2_, and (C) WO_3_/TiO_2_ nanocomposites.

In the core–shell nanocomposites, the photoinduced charges were considered to be adequately separated. Because the conduction band (CB) of TiO_2_ has a more negative redox potential (−0.5 V) than the CB of WO_3_ (+0.2 V), as reported by Escobar *et al.* (2020),^38^ the photogenerated electrons in TiO_2_ can easily be transferred to WO_3_ to reduce W^6+^ to W^5+^.^48^ According to Escobar *et al.* (2020), the valence band (VB) of WO_3_ has a more positive redox potential (+3.1 V) than the VB of TiO_2_ (+2.8 V);^38^ therefore, the photogenerated h^+^ can move from WO_3_ to TiO_2_ (Fig. 8). This charge separation extended the lifetime of the active species and improved the activity of holes on TiO_2_@WO_3_, while WO_3_ rapidly caused recombination and decreased photocatalytic activity, although it had the highest *K*_L_ value (Tables 2 and 3). In the codeposited WO_3_/TiO_2_, similar charge separation occurred, and both holes and photoelectrons were active on the surface. In the reversed core–shell WO_3_@TiO_2_, the photoexcited electrons (and superoxide anions) were likely to be the main species, but the electron transfer process from TiO_2_ to WO_3_ was competitive with the process of superoxide anion generation. This competition also occurred for hole transfer, and therefore, the activity of WO_3_@TiO_2_ was strongly suppressed. These mechanisms are illustrated in Fig. 8.

## 4. Conclusions

In this study, WO_3_-loaded TiO_2_ nanocomposites of various WO_3_ compositions were prepared using sol–gel (coprecipitation) and hydrothermal (core–shell) approaches. The structures of the nanocomposites were analyzed using XRD, SEM, EDX, XPS, and DRS, and adsorption-driven photocatalysis was comprehensively examined. Using cationic methylene blue (MB^+^) and anionic methyl orange (MO^−^), the adsorption behaviors of the dyes were explained by the electrostatic interaction between the dyes and the negatively charged surfaces of metal oxides, especially with respect to the tungsten component. From the Langmuir adsorption model, the adsorption rate and the adsorption capacity were analyzed for each metal oxide. The core–shell TiO_2_@WO_3_ demonstrated greater maximum adsorption capacity (*q*_m_ = 55.8 mg g^−1^) than the sol–gel-produced nanomaterials, indicating that more active sites were available for photocatalysis. Through a kinetic study, the photocatalytic decomposition reactions of both MB^+^ and MO^−^ on the sol–gel and core–shell metal oxides were analyzed. The WO_3_-loaded TiO_2_ nanocomposites showed considerably higher activity for MB^+^ than for MO^−^. The reaction rate per (adsorption rate x adsorption capacity) was calculated for each photocatalyst, and a synergistic effect was found. Using scavengers for active species, a charge separation mechanism was considered to improve the photocatalytic activity of complex metal oxides relative to simple oxides. Thus, the high efficiency of the core–shell TiO_2_@WO_3_ was explained. These results and the approaches used in this study could be useful for designing photocatalysts consisting of hybrid metal oxides.

## Author contributions

Abdisa Habtamu: investigation, formal analysis, writing-original draft, validation. Masaki Ujihara: conceptualization, methodology, project administration, supervision, resources, writing-review and editing, visualization, validation.

## Conflicts of interest

The authors disclaim any conflicts of interest.

## Supplementary Material

RA-013-D3RA01582C-s001
