# Supplementary material for: The mechanism of water pollutant photodegradation by mixed and core–shell WO3/TiO2 nanocomposites
Source: RSC Adv. 2023 Apr 25;13(19):12926–40. doi: 10.1039/d3ra01582c (PMC10128107; doi:10.1039/d3ra01582c)
Supplement: RA-013-D3RA01582C-s001 [file RA-013-D3RA01582C-s001.pdf]

Electronic Supplementary Material (ESI) for RSC Advances.
This journal is © The Royal Society of Chemistry 2023

Mechanism of Water Pollutant Photodegradation by Mixed and Core-Shell WO_3/TiO_2 Nanocomposites

Abdisa Habtamu [a] and Masaki Ujihara *[b]

[a] *Graduate Institute of Applied Science and Technology, National Taiwan University of Science and Technology, 43 Keelung Road, 10607, Taipei, Taiwan.*
[b] *Graduate Institute of Applied Science and Technology, National Taiwan University of Science and Technology, 43 Keelung Road, 10607, Taipei, Taiwan. E-mail: masaki.ujihara@mail.ntust.edu.tw*

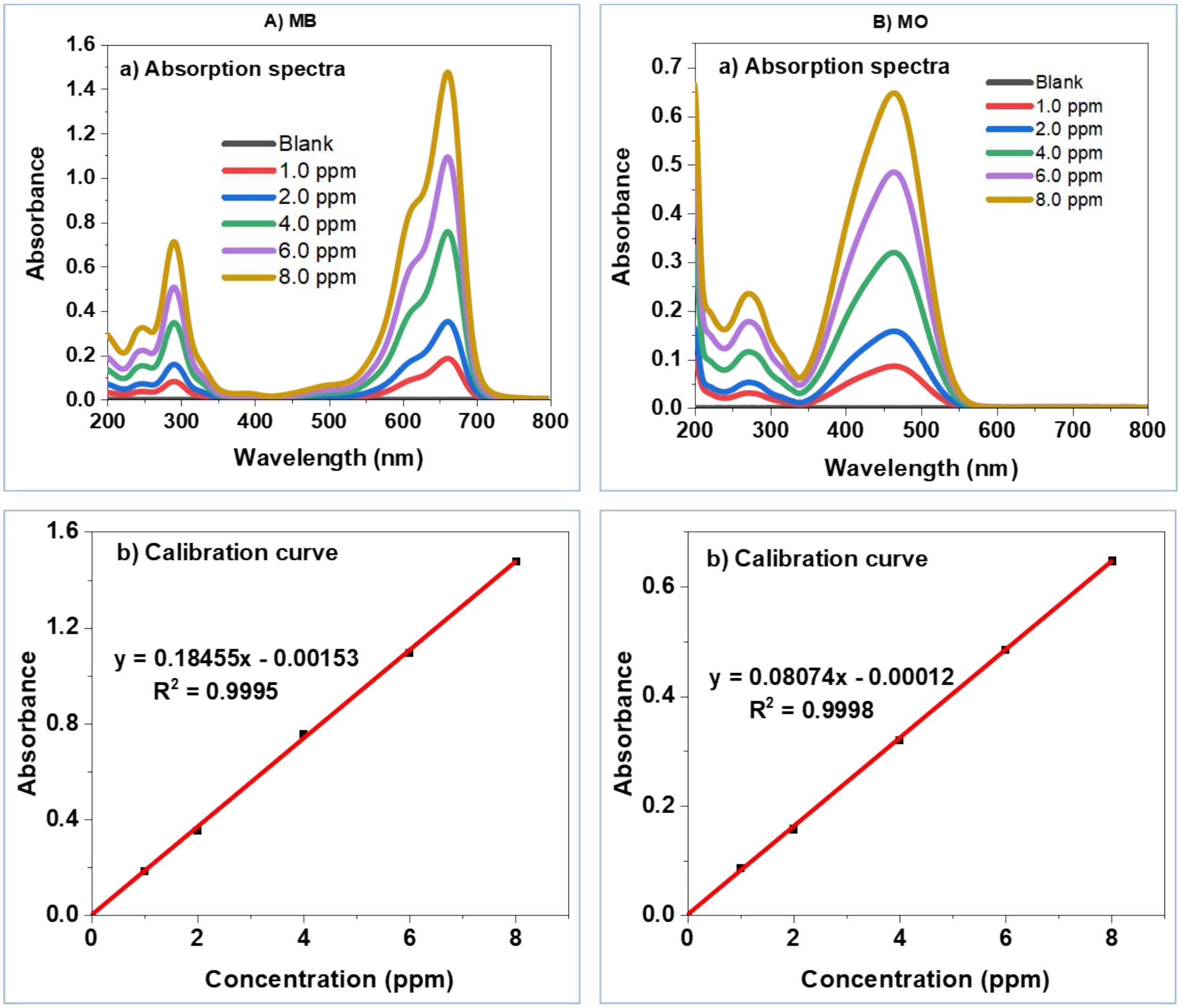

Figure S1. UV-vis absorption spectra and calibration curves for (A) MB^+ at 662 nm and (B) 464 nm for MO^-.

Table S1 O 1s peak parameters and % area for each composite.

	**Peak Type**	**Position (eV)**	**FWHM (eV)**	**Area**	**%Area**
5 wt% WO_3/TiO_2	Peak 1	530.35	1.242	501.23	68.81
	Peak 2	531.76	1.6	111.29	15.28
	Peak 3	533.42	1.81	115.92	15.91
8 wt% WO_3/TiO_2	Peak 1	529.68	1.3	576.11	72.23
	Peak 2	530.94	1.63	144.13	18.08
	Peak 3	532.75	1.54	77.04	9.668
10 wt% WO_3/TiO_2	Peak 1	529.98	1.26	622.56	71.85
	Peak 2	531.28	1.47	152.49	17.60
	Peak 3	533.01	1.405	91.47	10.56
$TiO_2@WO_3$	Peak 1	530.00	1.13	477.41	75.15
	Peak 2	531.00	1.37	111.04	17.48
	Peak 3	532.70	1.33	46.86	7.38
$WO_3@TiO_2$	Peak 1	530.00	1.211	522.03	77.76
	Peak 2	531.32	1.15	74.14	11.04
	Peak 3	532.48	1.384	75.16	11.20

Table S2 Ti 2p peak parameters and % area for each composite.

	Peak Type	Position (eV)	FWHM (eV)	Area	%Area
5 wt% WO_3/TiO_2	Ti $2p_{3/2}$	459.1	1.17	424.14	64.05
	Ti $2p_{1/2}$	464.77	2.11	238.04	35.95
8 wt% WO_3/TiO_2	Ti $2p_{3/2}$	458.35	1.326	457.53	71.32
	Ti $2p_{1/2}$	464.05	2.155	183.96	28.68
10 wt% WO_3/TiO_2	Ti $2p_{3/2}$	458.92	1.15	462.72	65.34
	Ti $2p_{1/2}$	464.62	2.17	245.48	34.66
TiO_2@WO_3	Ti $2p_{3/2}$	459.25	1.154759	467.95	66.99
	Ti $2p_{1/2}$	464.93	2.089	230.5865	33.01
WO_3@TiO_2	Ti $2p_{3/2}$	458.73	1.1	467.6004	69.18
	Ti $2p_{1/2}$	464.42	2.05	208.3091	30.82

Table S3 W 4f peak parameters and % area for each composite.

	Peak Type	Position (eV)	FWHM (eV)	Area	%Area	W^{5+}/W^{6+} Ratio	Ti 2p/W 4f Ratio
5 wt% WO_3/TiO_2	W^{5+} $4f_{7/2}$	35.56	1.123	46.97	32.80	3.09	4.62
	W^{5+} $4f_{5/2}$	37.44	1.206	61.24	42.77		
	W^{6+} $4f_{7/2}$	36.48	0.779	15.71	10.97		
	W^{6+} $4f_{5/2}$	38.25	1.106	19.27	13.46		
8 wt% WO_3/TiO_2	W^{5+} $4f_{7/2}$	34.97	1.45	72.68	39.77	2.42	3.51
	W^{5+} $4f_{5/2}$	36.9	1.14	56.70	31.03		
	W^{6+} $4f_{7/2}$	36.06	0.91	20.41	11.17		
	W^{6+} $4f_{5/2}$	37.6	1.42	32.94	18.03		
10 wt% WO_3/TiO_2	W^{5+} $4f_{7/2}$	35.05	1.68	123.9	53.61	4.59	3.06
	W^{5+} $4f_{5/2}$	36.95	1.07	65.89	28.50		
	W^{6+} $4f_{7/2}$	36.35	0.75	13.43	5.81		
	W^{6+} $4f_{5/2}$	37.54	1.12	27.95	12.09		
$TiO_2@WO_3$	W^{5+} $4f_{7/2}$	35.31	1.1	50.21	27.46	2.03	3.82
	W^{5+} $4f_{5/2}$	37.45	1.25	72.37	39.57		
	W^{6+} $4f_{7/2}$	36.56	1.11	35.10	19.19		
	W^{6+} $4f_{5/2}$	38.74	1.21	25.20	13.78		
$WO_3@TiO_2$	W^{5+} $4f_{7/2}$	35.2	1.23	45.46	33.70	13.40	5.00
	W^{5+} $4f_{5/2}$	37.12	1.443	80.09	59.36		
	W^{6+} $4f_{7/2}$	36.36	0.65	4.83	3.58		
	W^{6+} $4f_{5/2}$	38.8	0.941	4.54	3.36		

Table S4 Comparison of photocatalysis performance for MB degradation

Catalyst	Preparation method	Light source	Degradation rate	Rate constant	Reference
TiO_2	Hydrothermal	Xe lamp, 300 W	75% in 240 min	0.00554 min^{-1}	W. Wang et al. 2019 [46]
Core-shell $WO_3@TiO_2$ (36 wt% of WO_3)	Sol-gel		100% in 240 min	0.01485 min^{-1} 2.68 times > TiO_2	
TiO_2	Sol-gel	Halogen lamp, 400 W	75% in 180 min	0.009 min^{-1}	*W. A. El-Yazeed & Ahmed, 2019
10 wt% WO_3/TiO_2 (mixed)			99% in 180 min	0.017 min^{-1} 2 times > TiO_2	
TiO_2	Hydrothermal	Xe lamp (350 W with 420 nm cut-off filter)	40.7% in 150 min	-	Q. Wang et al. 2021 [48]
10 wt% WO_3/TiO_2 (mixed)			87.8% in 150 min	-	
TiO_2	Precipitation	Halogen lamp, 500 W	12% in 60 min	0.001 min^{-1}	M. F. Mubarak et al. 2022 [65]
Core-shell $TiO_2@CoFe_3O_4$	Co-precipitation		91% in 60 min	0.016 min^{-1} 16 times > TiO_2	
TiO_2	As-purchased	UV light (365 nm)	80% in 120 min	0.0117 min^{-1}	R. Wahyuono et al. 2019 [51]
25 wt% WO_3/TiO_2 (mixed)	Sol-gel		92% in 120 min	0.0185 min^{-1} 1.58 times > TiO_2	
TiO_2	Sol-gel	UV LED (365 nm)	24.3% in 120 min	0.0023 min^{-1}	This work
8 wt% WO_3/TiO_2 (mixed)			94.9% in 120 min	0.0248 min^{-1} 10.78 times > TiO_2	
Core-shell $TiO_2@WO_3$ (10 wt% WO_3)	Hydrothermal		95.8% in 60 min	0.0533 min^{-1} 23.17 times > TiO_2	
Core-shell $WO_3@TiO_2$ (10 wt% WO_3)			82.5% in 120 min	0.0141 min^{-1} 6.13 times > TiO_2	

* W. A. El-Yazeed and A. I. Ahmed, *Inorganic Chemistry Communications*, 2019, **105**, 102-111.

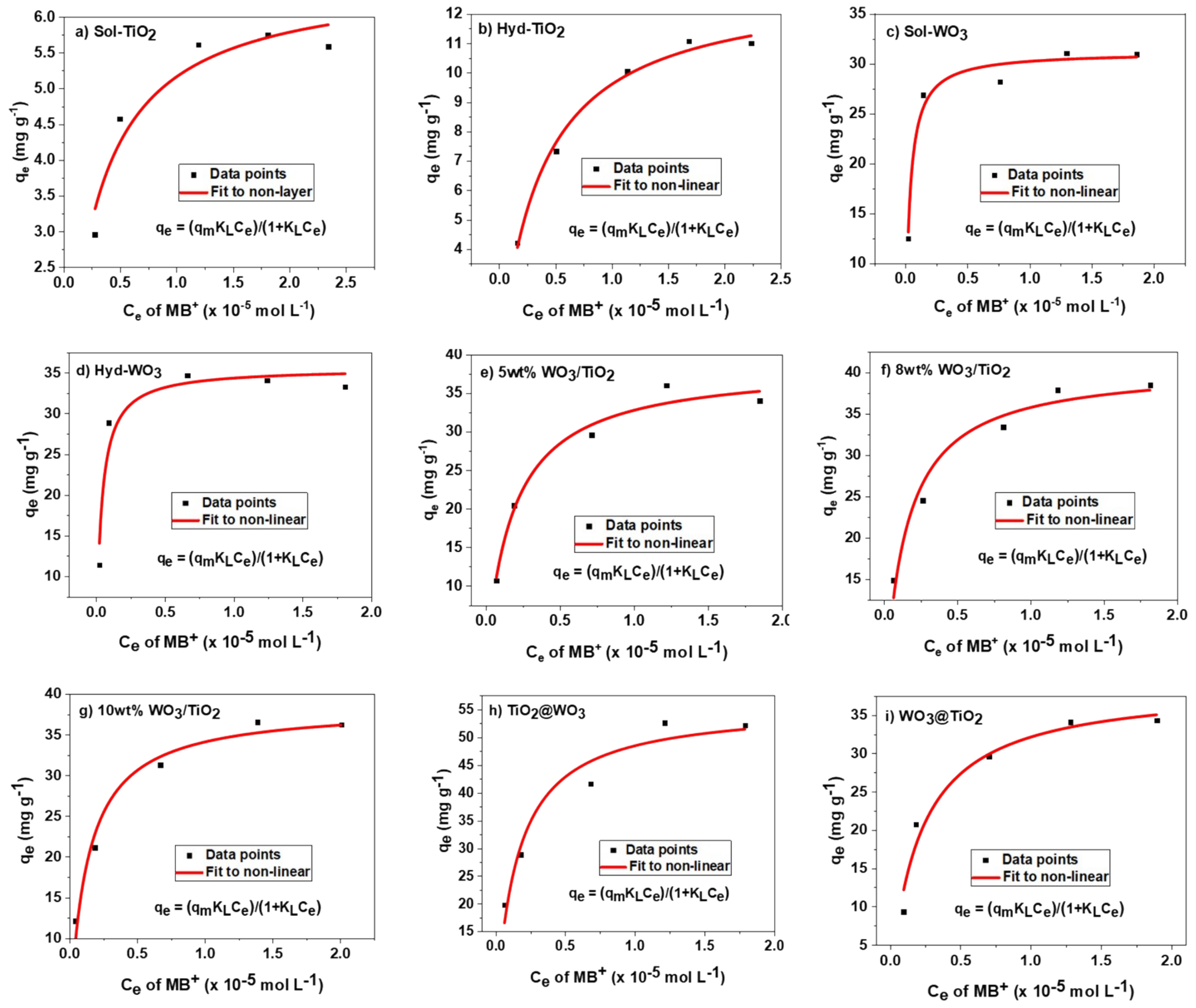

Figure S2. Adsorption behaviors of MB^+ onto nanocomposites and non-linear Langmuir isotherm fitting.

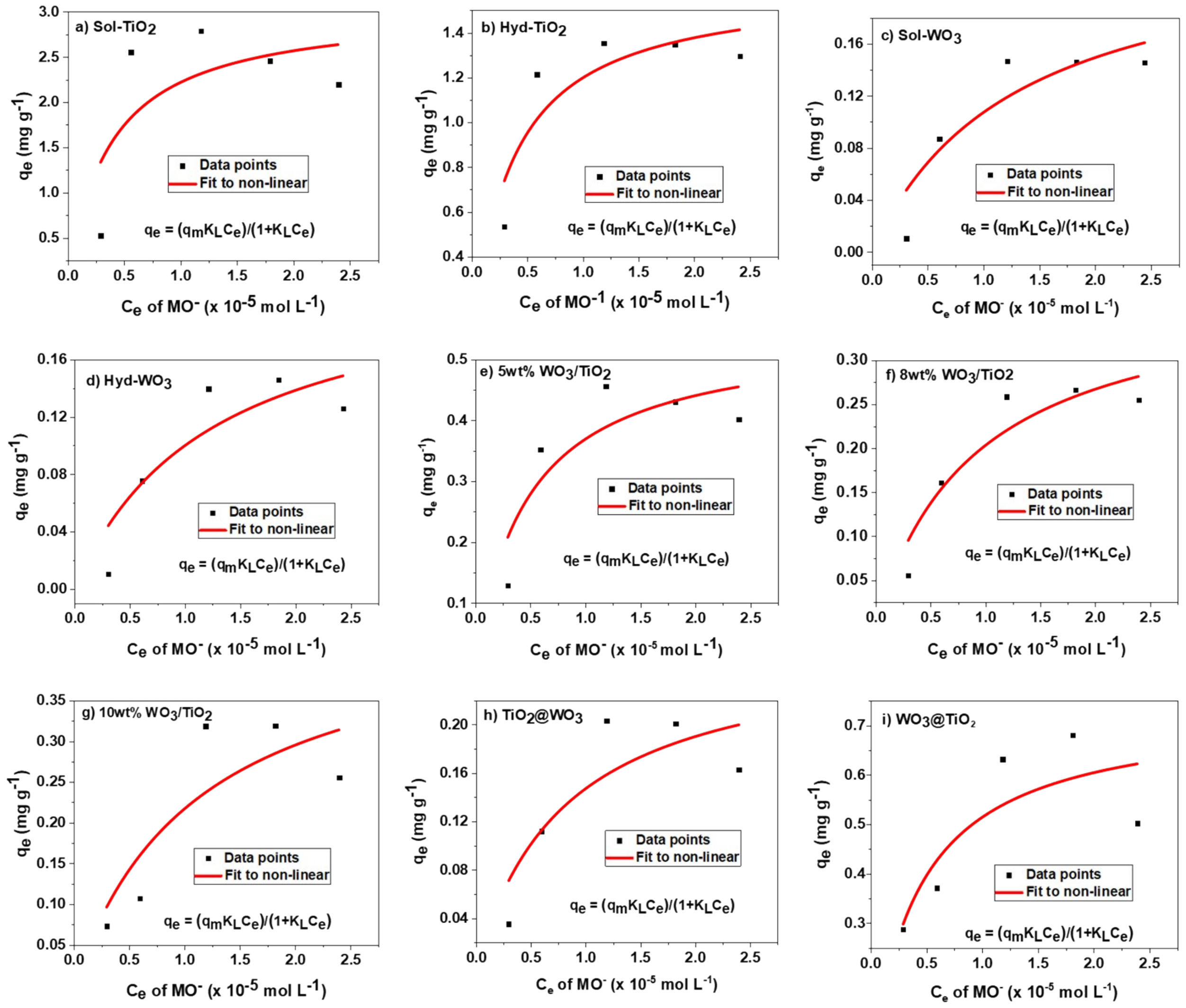

Figure S3. Adsorption behaviors of MO^- onto nanocomposites and non-linear Langmuir isotherm fitting.

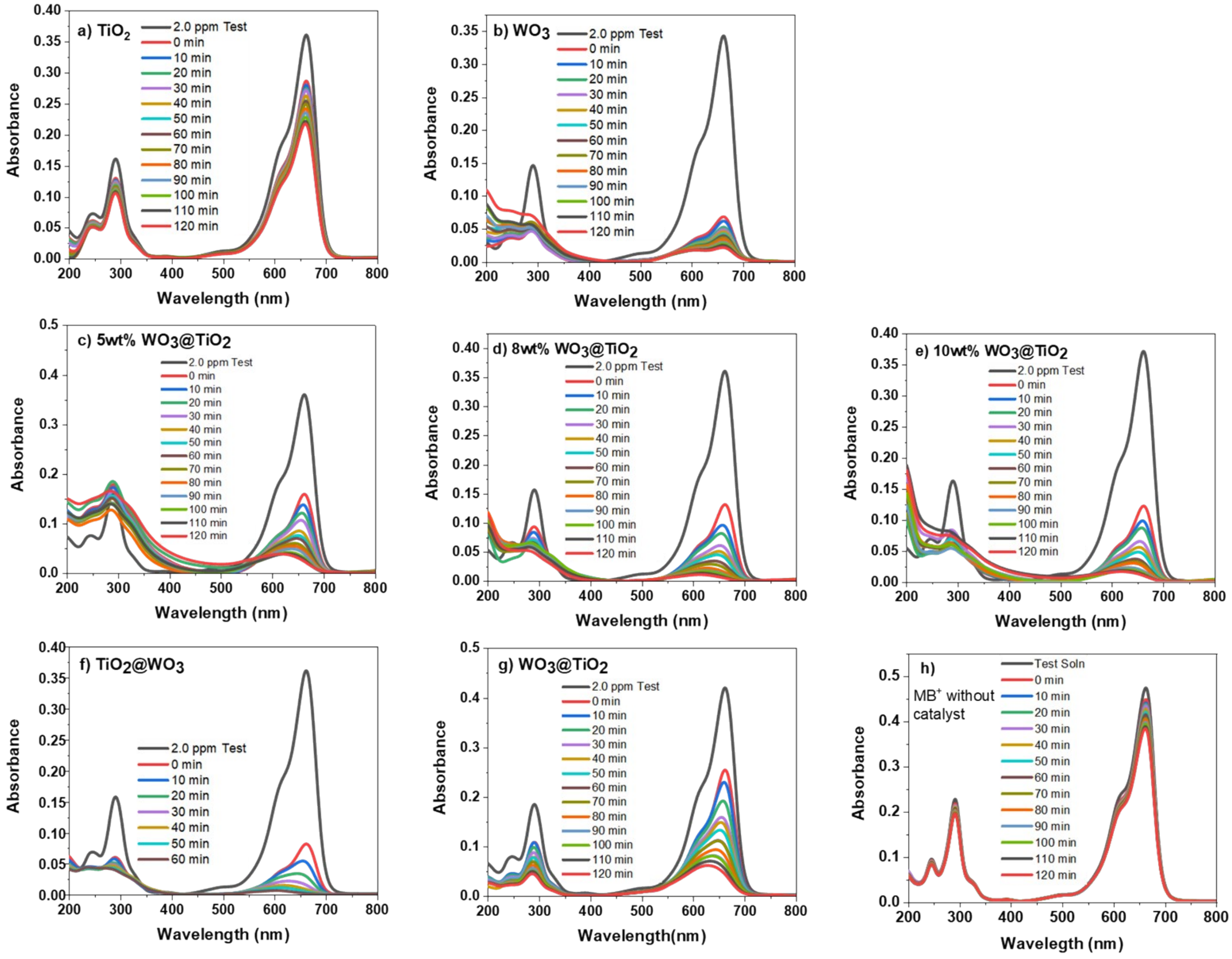

Figure S4. Absorption spectra of MB^+ with various nanocomposites under UV light and without nanocomposite under UV light.

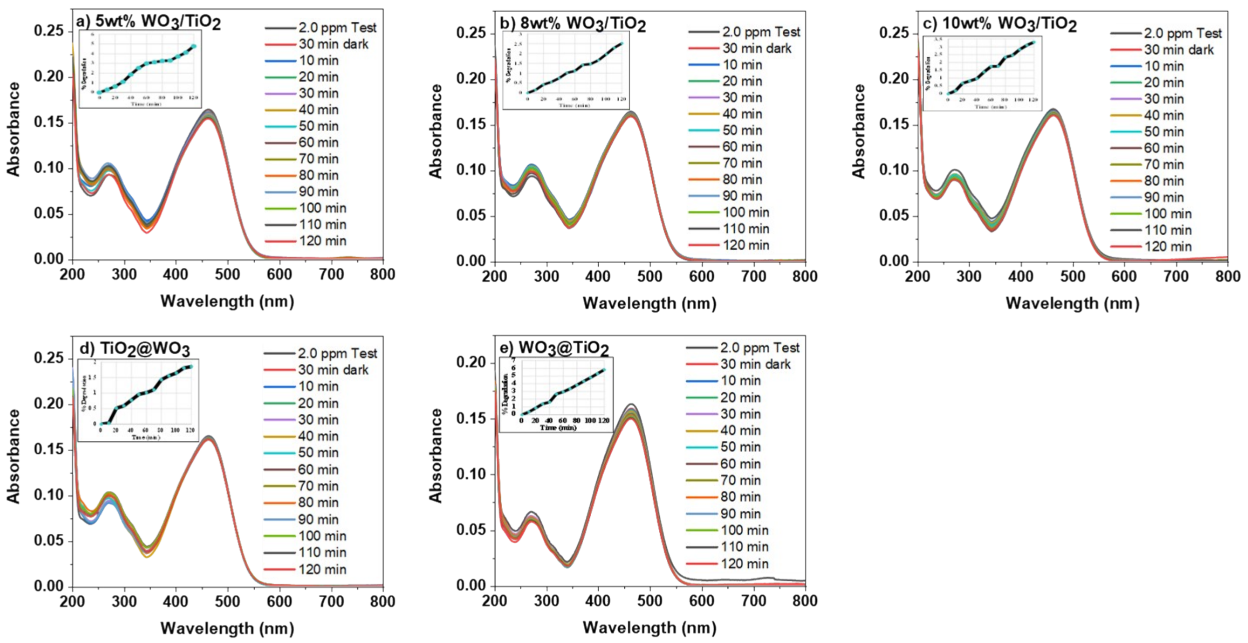

Figure S5. Absorption spectra of MO^- with various nanocomposites under UV light and without nanocomposite under UV light.
